# Antibiotic Resistance Pattern of Extended Spectrum Beta Lactamase Producing *Escherichia coli* Isolated From Patients With Urinary Tract Infection in Morocco

**DOI:** 10.3389/fcimb.2021.720701

**Published:** 2021-08-18

**Authors:** Mohamed Kettani Halabi, Fatima Azzahra Lahlou, Idrissa Diawara, Younes El Adouzi, Rabiaa Marnaoui, Rachid Benmessaoud, Imane Smyej

**Affiliations:** ^1^Faculty of Medicine, Mohammed VI University of Health Sciences (UM6SS), Casablanca, Morocco; ^2^National Reference Laboratory, Mohammed VI University of Health Sciences (UM6SS), Casablanca, Morocco; ^3^Faculty of Nursing and Allied Health Sciences, Mohammed VI University of Health Sciences (UM6SS), Casablanca, Morocco; ^4^Faculty of Pharmacy, Mohammed VI University of Health Sciences (UM6SS), Casablanca, Morocco

**Keywords:** antibiotic resistance, ESBL, *Escherichia coli*, urinary tract infection, multidrug resistant

## Abstract

Extended-spectrum β-lactamases producing *Escherichia coli* (ESBL-EC) lend resistance to most β-lactam antibiotics. Because of limited treatment options, ESBL-EC infections are generally more difficult to treat, leading to higher hospital costs, reduced rates of microbiological and clinical responses, and a threat to the patient’s life. This study aimed to determine the antibiotic resistance pattern of ESBL-EC isolated from patients with urinary tract infection in Morocco. This retrospective laboratory-based study was conducted at Cheikh Khalifa International University Hospital, Casablanca, from January 2016 to June 2019. A total of 670 urine samples were collected from urinary tract infection patients and processed by standard microbiological methods. *In vitro* susceptibility testing to different antibiotics of all identified isolates of *Escherichia coli (E. coli)* was performed following Kirby–Bauer’s disc diffusion method on Mueller–Hinton Agar according to the EUCAST standards. The reviewing of ESBL-EC was confirmed by the appearance of a characteristically shaped zone referred to as a “champagne cork” using the Combined Disk Test. Among a total of 438 *E. coli* isolated from nonrepetitive urine samples, two hundred fifty-nine (59%) were ESBL-EC, of which 200 (77%) were isolated from adult patients (over the age of 50) and the majority were female. All ESBL-EC isolates were resistant to third-generation cephalosporin and quinolones and sensitive to carbapenem and fosfomycin. Knowledge of antimicrobial resistance patterns in ESBL-EC, the major pathogen associated with urinary tract infection, is indispensable as a guide in choosing empirical antimicrobial treatment.

## Introduction

*Escherichia coli* as an *Enterobacteriaceae* member is the most common causative bacteria associated with urinary tract infections up to 80% (Farzana et al., 2013; Shakya et al., 2017). β-Lactam antibiotics are the safest and most frequently prescribed antimicrobial agents for urinary tract infection (UTI) (Magale et al., 2016). They are a class of broad-spectrum antibiotics that contain a beta-lactam ring in their molecular structure (Ahmed et al., 2015; Rajabnia et al., 2019). Several studies prove the increased resistance of *E. coli* in UTI to beta-lactam antibiotics (Glasner et al., 2013; Tacconelli et al., 2018). Another worry is the “emergence of extended spectrum Beta-Lactamase (ESBL) producing bacteria” that hydrolyze beta-lactam ring, inactivate the antibiotic, and reduce consequently the number of treatment options (Ruano-de-pablo & Losada-pinedo, 2016; Rajabnia et al., 2019).

The first bacteria expressing acquired ESBL were identified in 1983 in Germany, and since 2000, ESBL-producing *Escherichia coli* (ESBL-EC) have spread throughout the world in both community and hospital settings (Kruglova & Stasheva, 2010; Rossignol et al., 2017; Rajabnia et al., 2019). Worryingly, the incidence of ESBL-EC continues to increase worldwide (Khanfar et al., 2009; Shakya et al., 2017). This situation leads to longer hospital stays, a dramatic increase in the consumption of antibiotics and consequently higher hospital expenses, and a trend toward higher mortality (Degnan et al., 2015; Cheng et al., 2016; Fernando et al., 2017). The beta-lactam antibiotics, in particular carbapenems, have been widely regarded as the treatment of choice for ESBL-EC infections (Nepal et al., 2017). However, the global emergence of beta-lactam antibiotic resistance strains of *enterobacteriacae* threatens the efficacy of this family of antibiotics (Stapleton et al., 2017; van Driel et al., 2019). Furthermore, frequent coresistance to sulfonamides and fluoroquinolones limits the availability of other therapeutic track (Cheng et al., 2016; Senard et al., 2020).

In Morocco, the ESBL-EC and its antibiotic susceptibility patterns have been very little studied. This kind of study would facilitate the understanding by clinicians of the therapeutic failure of the conventional antibiotic therapies used in serious infections caused by ESBL-EC and would guide them to adopt an appropriate empirical antibiotic therapy. In this perspective, we conducted a laboratory-based study in order to identify the ESBL-EC isolated from a patient with urinary tract infection and its phenotypic profiles of antibiotic susceptibility. The evolution of the ESBL-EC and some risk factors were also explored.

## Methods

### Study Design and Data Collection

This is a retrospective laboratory-based study of all isolates of ESBL-EC isolated from urine samples at the National Reference Laboratory (LNR) of Cheikh Khalifa International University Hospital (HCK) from January 2016 to June 2019. Patient data were retrieved from the bacteriology service information system of the LNR.

Patients with clinically suspected UTI were included in the study. We defined a significant monomicrobic bacteriuria as the culture of a single bacterial species from the urine sample at a concentration of >10^5^ CFU/ml (urine bag). Patients with multimicrobial urine samples or isolates collected >72 h after hospitalization were excluded. We also excluded patients who were on antibiotics, patients who had hospital admission within the last 3 months, or patients on urinary catheters because we wanted to exclude recurrences due to partially treated urinary tract infections and healthcare-associated infections.

A total of 670 urine samples were included in this study; only the first isolate of *E. coli* from each patient was considered to avoid overestimation of resistance rates from different urine specimens. Each isolate was classified as being of community or hospital origin. Community samples included those originating from outpatient rooms of the hospital or of external general practitioners in the surrounding area. Hospital samples were provided both from the emergency department and hospital departments.

The collected samples according to current European recommendations were labeled properly and were immediately delivered to the LNR for further processing. When immediate delivery was not possible, the specimens were refrigerated at 4°C to 6°C (Cornaglia et al., 2012).

### Laboratory Examinations of Samples

All urine samples were cultured on routine culture media by semiquantitative method as described in the World Health Organization (WHO) manual (Vandepitte et al., 2003). In short, 1 μl of urine (by using a calibrated wire loop) was inoculated on CLED (Oxoid Company, Britain) and blood agar plate (BioMèrieux, Lyon, France) by streaking and incubated aerobically for 24 h at 37°C. Isolates were isolated and identified depending on their morphology in Gram’s staining, cultural characteristics, and biochemical properties, as per the European Manual of Clinical Microbiology (Cornaglia et al., 2012).

### Antimicrobial Susceptibility Testing

*In vitro* susceptibility testing to different antibiotics of all identified isolates of *E. coli* was performed following Kirby–Bauer’s disc diffusion method on Mueller–Hinton Agar (Hi-Media, India) according to the European Committee on Antimicrobial Susceptibility Testing (EUCAST) criteria version 6.0 (EUCAST, 2016). Antibiotics tested in our study include the following: amoxicillin (AMX; 10 μg), amoxicillin clavulanate (AMC; 20/10 μg), cefoxitin (FOX; 30 μg), piperacillin/tazobactam (TZP; 30/6 μg), ticarcillin (TIC; 85 μg), fosfomycin (FOS; 200 μg), gentamycin (GEN; 10 μg), tobramycin (TOB; 10 μg), amikacin (AN; 30 μg), norfloxacin (NX; 10 μg), cefalexin (CN; 30 μg), ceftriaxone (CTR; 30 μg), ceftazidime (CAZ; 30 μg), cefixime (CFM; 10 μg), cefotaxime (CTX; 30 μg), imipenem (IPM; 10 μg), ertapenem (ETP; 10 μg), co-trimoxazole (SXT; 1.25/23.75 μg) (Hi-Media, India), and colistin (CT; 10 μg) (Oxoid™, UK). The clinical interpretation (S/I/R) of results took into consideration diameters or breakpoints recommended by the EUCAST (2016). An isolate was considered as multidrug-resistant if it was resistant to ≥3 groups of antibiotics.

### Screening and Confirmation of ESBL Producers

ESBL production was determined by the double-disk diffusion method, following the criteria established by the CA-SFM/EUCAST (EUCAST, 2016; Dubreuil et al., 2018). The presence of ESBL was concluded when the inhibition zone around cefotaxime (30 μg), ceftazidime (30 μg), cefepime (10 μg), or aztreonam (30 μg) antibiotic disks was enhanced on the side of the clavulanate-containing disk, resulting in a characteristically shaped zone referred to as a “champagne cork” (Garrec et al., 2011; van Driel et al., 2019; Senard et al., 2020). This method was carried out on Mueller Hinton Agar (Hi-Media, India), which was inoculated with the test strain and then incubated in ambient air for 16–18 h of incubation at 35 ± 2°C.

### Quality Control

Each batch of media and reagents underwent sterility and performance testing. During antibiotic susceptibility test, quality control was performed using the control strains of *E. coli* ATCC 25922 (Hansen et al., 2014; Nellums et al., 2018).

### Statistical Analysis

Statistical data analysis was performed using the chi-square test for testing relationships between categorical variables. Data were seized and analyzed by using IBM SPSS Statistics for Macintosh, Version 23.0 (IBM Corp, Armonk, NY, USA) and presented in percentage base distribution. Descriptive statistical analysis (including means and percentages to characterize data) was performed using Microsoft Excel, Version 16.16.21 (Redmond, WA, USA, Microsoft Corp.). All P values were two-sided, and P < 0.05 was considered statistically significant.

## Results

**Figure 1:**
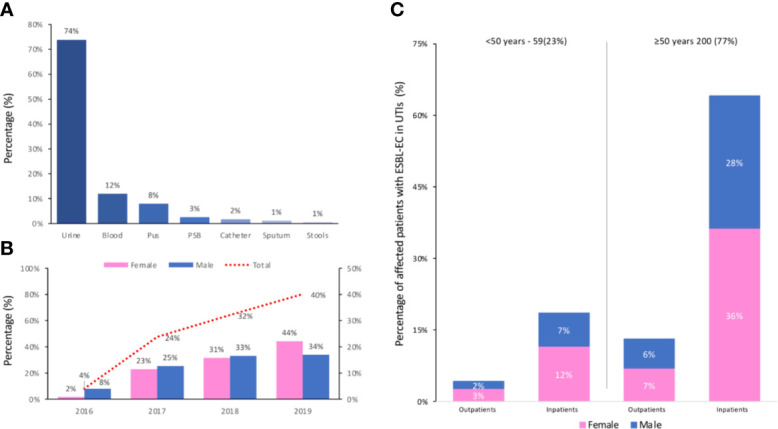
**(A)** Prevalence of ESBL-EC from different clinical specimens; Protected specimen brush (PSB). **(B)** Evolution of urinary tract infections due to ESBL-EC (Male, Female and Total) per year (from 2016 to 2019). **(C)** Age distribution of the affected patients with ESBL-EC (Male/Female) in the outpatient and inpatient groups.

During the study period, 670 urine samples meeting the inclusion criteria were collected from urinary tract infections from patients in different departments of the hospital (HCK). A total of 438 (65%) *E. coli* isolates were isolated from nonrepetitive urinary tract infections (UTIs) of which 259 (59%) were ESBL-EC. The prevalence study of ESBL-EC isolates from different clinical specimens was in favor of predominance in urine up to 74% (n=259) compared to other clinical samples (**Figure 1A**). Monitoring of these urinary tract infections due to ESBL-EC showed an upward trend, expressed more in women than in men, with an increase between 2016 and the end of 2019 from 2% to 44% in women and 8% to 34% in men, thus reaching a total increase from 4% to 40% (**Figure 1B**). In other words, out of 259 samples of ESBL-EC, 149 (58%) samples were of female patients and 110 (42%) were of male patients (**Table 1**). Approximately one-third of the patients had at least one comorbid disease.

**Table 1:** Analysis of some risk factors in urinary *E. coli* isolates from 2016 to 2019.

	Total	Non-ESBL *E. coli*	ESBL producing *E. coli*	P^*^
	N = 438	N = 179	N = 259	
**Sex**				
Male	162 (37%)	52 (29%)	110 (42%)	.004
Female	276 (63%)	127 (71%)	149 (58%)	
**Age**				
<50	123 (28%)	64 (35%)	59 (22%)	.003
0–10	32 (6%)	18 (10%)	14 (6%)	
11–20	7(2%)	7 (4%)	0 (0%)	
21–30	17(4%)	17 (9%)	0 (0%)	
31–40	34 (8%)	13 (7%)	21 (8%)	
41–50	33 (8%)	9 (5%)	24 (9%)	
>50	315 (72%)	115 (65%)	200 (77%)	
**Origin**				
Inpatients	356 (81%)	142 (79%)	214 (83%)	.385
Outpatients	82 (19%)	37 (21%)	45 (17%)	

^P*: “p value” obtained by the chi-square test provides to determine the existence of link between ESBL-EC and various risk factors.^

The results of univariate analysis showed that patients’ gender and age (patients over the age of 50) were significantly associated with UTI due to ESBL-producing isolates (**Table 1**). Female gender was associated with increased risk of being infected by ESBL-EC compared to male (p = 0.004). In addition, patients over the age of 50 were found to have a higher risk of being infected by ESBL-EC compared to adults (p = 0.001).

Furthermore, the calculated p-value (p = 0.385) as displayed in **Table 1** shows that the risk of contracting ESBL-EC is similar between the two groups studied (inpatients/outpatients). Among the affected patients with ESBL-EC, the age groups over 50 years of age (outpatients: 17%; inpatients: 83%) were the most numerous. In this age group, the female-to-male ratio was 1.25 (53% female) in the outpatient group, while in the inpatient group, this value was 1.38 (57% female). The age distribution of the affected patients with ESBL-EC (male/female) in the outpatient and inpatient groups is presented in **Figure 1C**.

The mean prevalence of UTI due to BLSE-EC in hospitalized patients during the study period (2016 to early 2019) was 66 (66%), and no significant difference per year was observed (data not shown).

The resistances observed in ESBL-EC sample are detailed in **Table 2**.

**Table 2:** Resistances among ESBL-producing *E. coli* isolates.

Antibiotic	Resistance	Susceptibility	Intermediate
**Beta-lactam family**			
Amoxicillin (AMX)	252 (100%)	1 (0%)	0
Amoxicillin/clavulanic acid (AMC)	171 (67%)	83 (33%)	0
Piperacillin/tazobactam (TZP)	97 (40%)	115 (47%)	32 (13%)
Ticarcillin (TIC)	176 (98%)	4 (2%)	0
Cefoxitin (FOX)	25 (10%)	212 (88%)	3 (2%)
Ceftazidime (CAZ)	225 (90%)	18 (7%)	8 (3%)
Cefotaxime (CTX)	208 (91%)	8 (3%)	13 (6%)
Cefixime (CFM)	231 (96%)	7 (3%)	2 (1%)
Imipenem (IPM)	7 (3%)	218 (96%)	2 (1%)
Ertapenem (ETP)	2 (1%)	199 (98%)	2 (1%)
**Others antibiotic family**			
Gentamicin (GM)	102 (41%)	140 (56%)	7 (3%)
Tobramycin (TM)	183 (73%)	57 (23%)	10 (4%)
Amikacin (AN)	46 (19%)	162 (66%)	37 (15%)
Norfloxacin (NX)	231 (93%)	14 (6%)	4 (1%)
Trimethoprim/sulfamethoxazole (SXT)	184 (80%)	45 (20%)	0
Fosfomycin (FOS)	5 (2%)	235 (98%)	0
Colistin (CT/CL)	1 (0%)	223 (100%)	0

Analysis of the β-lactam family shows that all strains of *E. coli* producing ESBLs are resistant to amoxicillin (AMX), 98% of strains are resistant to ticarcillin (TIC), and from 90% to 96% of strains are resistant to third-generation cephalosporins. Penicillin protected by beta-lactamase inhibitors such as piperacillin/tazobactam (TZP) and amoxicillin/clavulanic acid is no longer effective against ESBL-EC with resistance ranging from 40% to 67%. However, the analysis shows that the carbapenems are very effective against ESBL-EC with a sensitivity ranging from 96% for imipenem to 98% for ertapenem.

**Table 2** shows also that ESBL-EC resistance affects other families of antibiotics with 93% resistance to quinolones (NX), 80% to sulfonamides (SXT), and 73% to tobramycin (TM). At the opposite, all ESBL-EC strains are sensitive to fosfomycin.

Moreover, all ESBL-EC were resistant either to at least two antibiotics belonging to two different families or to the protected penicillins (**Table 3**). This same table also shows that in the category of multidrug resistant, 47% of ESBL-EC are resistant to penicillin, protected penicillin, third-generation cephalosporins, and quinolones. It also shows that the total amount of multidrug-resistant isolates among ESBL-CE was 1% with resistance to cephalosporins (second and third generation), amoxicillin, combinations of antibiotics penicillin/beta-lactamase inhibitor, norfloxacin, tobramycin, gentamicin, and sulfamethazole/trimethoprim.

**Table 3:** Multidrug resistant profile of ESBL-EC isolates.

Resistance profile	No. of isolates with resistance profile [N (%)]	Resistance category
AMC, AMX	150 (58%)	Drug resistant
CAZ, NX	167 (65%)	Drug resistant
AMC, NX, AMX	140 (54%)	Drug resistant
AMC, CAZ, NX, AMX	121 (47%)	Multidrug resistant
AMC, CAZ, TM, NX, AMX	101 (39%)	Multidrug resistant
AMC, CAZ, TM, GM, NX, AMX	58 (23%)	Multidrug resistant
AMC, CAZ, TM, GM, SXT, NX, AMX	48 (19%)	Multidrug resistant
AMC, FOX, CAZ, TM, GM, SXT, NX, AMX	19 (7%)	Multidrug resistant
AMC, FOX, CAZ, TM, GM, AN, SXT, NX, AMX	3 (1%)	Multidrug resistant

AMX, amoxicillin; AMC, amoxicillin/clavulanic acid; FOX, cefoxitin; NX, norfloxacin; CAZ, ceftazidime; GM, gentamicin; TM, tobramycin; AN, amikacin; SXT, trimethoprim/sulfamethoxazole.

## Discussion

The emergence and rapid dissemination of multidrug-resistant *Enterobacteriaceae* worries the whole world and, in particular, ESBL-producing enterobacteriaceae. Since the 2000s, ESBL-EC have been considered as serious pathogens both in nosocomial and community infections around the world, and their virulence varies by region (Shakya et al., 2017). Knowledge of the local epidemiology and its evolution over time is a key step in choosing the effective first-line antibiotic therapy adapted to each region. However, in our country, we are aware of the heavy burden we share due to the multitude of factors integrated in the characteristics of practice of medicine, policy, and the health system (Pokharel et al., 2019). Moreover, ESBL-EC pathogens are more common in females and in hospital settings (**Figure 1C**) and are for more than half of nosocomial origin, which is often very complicated to treat (Iqbal, 2019). ESBL-EC pose a major threat in the management of uropathogens and threaten the future activity of last-line molecules (Sbiti et al., 2017). In our study, more than two-thirds of the isolates (63%) were ESBL-EC. A high prevalence of ESBL-EC strains have been raised quite consistently from previous studies carried out in Morocco (Lahlou Amine et al., 2009; Romli et al., 2011; El Bouamri et al., 2014a; El bouamri et al., 2014b; Sbiti et al., 2017) and outside (Gravey et al., 2017; Critchley et al., 2019; Koguchi et al., 2020; Tan et al., 2020; van Hout et al., 2020).

In other words, 409 (61%) *Enterobacteriaceae* isolated from urine culture were ESBL-producing. Out of 438 isolates of *E. coli*, 259 (59%) are ESBL-EC and 179 (41%) are not ESBL-EC. A few other studies mentioned the prevalence of ESBL-EC isolates 37.11% (Rajabnia et al., 2019), 46.87% (Kulkarni et al., 2016), and 82.6% (Singh et al., 2016) respectively. These variations in the rate of ESBL-EC among UTI cases might be attributable to the local antibiotic prescribing policy, abuse of broad-spectrum antibiotics especially penicillin and third-generation cephalosporins, geographical difference, and hospitalization (Nepal et al., 2017; Parajuli et al., 2017). Furthermore, we did not observe a significant evolution in the rate of isolated ESBL-EC. Their incidence rate in urinary tract infection was 75%. However, an increase in the number of ESBL-EC is well described in different regions of the world (Fernando et al., 2017; Rajabnia et al., 2019; Rohde et al., 2020).

As seen in previous studies, sex, age, and hospitalization are proposed as risk factors (Lahlou Amine et al., 2009; Ahmed et al., 2015; Flammang et al., 2017). In the present study, among the positive *E. coli* cases, the prevalence rate of UTIs was found more in females, 276 (63%), than males, 172 (37%). As for positive ESBL-EC cases, the prevalence rate was 149 (58%) in females and 110 (42%) in males. Females are more frequently affected due to the anatomy of their genitals. In other words, the short length of the urethra and its closeness to the anus favor the proliferation of ESBL-EC (Tille, 2018). The patient’s sex is a risk factor of UTI (p= 0.004), which should be taken into account. In addition, the highest ESBL isolates were found in the age group over 50 years, including the prevalence of ESBL-EC organisms, which was above 77% (**Table 1**). The risk of ESBL-EC infection was significantly higher in the age group over 50 years than lower age groups (p < 0.003). Many studies confirm that advanced age (usually over 65 years) is a UTI risk factor for ESBL-EC (Heytens et al., 2017; Sbiti et al., 2017; van Driel et al., 2019). The reason for this may be due to the immunological status of these patients being more vulnerable to infections. Those under 50 years, including children, are not spared from this risk (Ruano-de-pablo & Losada-pinedo, 2016; Heytens et al., 2017). Likewise, our results on the prevalence of ESBL-EC in both the hospital (83%) and community (17%) settings are probably underestimated and drive us to suggest the need of systemic surveillance. The healthy carriers of ESBL-EC may contribute to the emergence of these bacteria in communities, which better explains the origin of the easy spread of resistances in a country, and the need of an appropriate national healthcare program.

According to the result of the antibiogram, the highest antibiotic resistance among ESBL-EC was observed with amoxicillin (100%), ticarcillin (98%), third-generation cephalosporin [cefixime (96%), cefotaxime (91%), and ceftazidime (90%)], and norfloxacin (93%). This study showed that ESBL-EC were resistant to third-generation cephalosporin and quinolones and sensitive to aminoglycoside and carbapenem. The highest antibiotic sensitivity was observed with colistin (100%), fosfomycin (98%), carbapenems [ertapenem (98%), imipenem (96%)], and cefoxitin (88%). Our findings are similar to some Moroccan studies (Lahlou Amine et al., 2009; El Bouamri et al., 2014a; El bouamri et al., 2014b; Sbiti et al., 2017) and others (Kulkarni et al., 2016; Ruano-de-pablo & Losada-pinedo, 2016; Tan et al., 2020) (**Table 2**).

We noted that many penicillins (combined with beta lactamase inhibitor) such as amoxicillin/clavulanic acid (67% resistance) or piperacillin/tazobactam (40%) used in practice as alternative antimicrobials were shown to be less effective against ESBL-EC. According to the standard guidelines, to use an antibiotic as an empirical antibiotic, it must have been tested >90% sensitive on the causative *E. coli* of that community (Bonnet et al., 2017). According to that criterion, only fosfomycin can be used as oral empirical antibiotics in our community. However, fosfomycin is currently unavailable in Morocco (Sbiti et al., 2017).

Moreover, several studies have concluded that the clinical picture of UTI cannot exactly predict whether it is ESBL UTI or not (Priyadharshana et al., 2019). The high resistance of ESBL-EC to many families of antibiotics reduced considerably the therapeutic options and maintains a continuous increase in the prescription of carbapenems (Lahlou Amine et al., 2009). Ertapenem, imipenem, doripenem, and meropenem are still the first choice of treatment for serious infections with ESBL-EC. It has been reported that >98% of the ESBL-EC, *K. pneumonia*, and *P. mirabilis* are still susceptible to these drugs (Fernando et al., 2017). But with the emergence of the carbapenem-resistant *Enterobacteriaceae*, some older drugs were found effective against ESBL-EC infections. Colistin and some aminoglycosides are considered effective in the treatment of carbapenem-resistant organisms (Fernando et al., 2017; Sbiti et al., 2017; Gajdács et al., 2019).

Senard et al. (2020), in a multicenter retrospective cohort study with propensity score analysis, showed that clinically and in terms of microbiological success, the efficacy of treatment with cefoxitin and carbapenems was similar in febrile UTIs due to ESBL-EC. However, cefoxitin is another antimicrobial drug missing from the Moroccan market.

## Conclusions

Today, the use of prophylactic antibiotics in UTIs due to ESBL-EC is no longer recommended because it favors emergence and spread of antibiotic-resistant strains (World Health Organization, 2018). In addition, inappropriate use of antibiotics has been shown to play a major role in the emergence of multidrug-resistant (MDR) bacteria (World Health Organization, 2018). This is the reason why clinicians should ensure the prescription and use of appropriate antibiotics during the recommended times and at controlled doses in order to prevent emergence of MDR such as ESBL-EC (**Table 3**) (Barguigua et al., 2019).

Some limitations need to be considered when interpreting the results of our study. First, the study was retrospective in nature and was primarily based on data from urinary isolates infected with *E. coli* previously collected at Cheikh Khalifa Hospital only, and with a maximum of one isolate per patient. Second, in spite of the lack of the data on ciprofloxacin resistance due to accessibility issues for computer databases, we believe that our results follow the same trend reported by previous Moroccan studies (Lahlou Amine et al., 2009; Romli et al., 2011; El Bouamri et al., 2014a; Sbiti et al., 2017). Third, the molecular evaluation of antibiotic resistance in isolates has not been studied, though it is possible that isolates carry resistance genes but do not express them phenotypically. And therefore, they can transmit this resistance to other bacteria. Finally, all of our isolates tested were ESBL-EC, results that are not necessarily applicable to other *Enterobacteriaceae*. Nevertheless, our results are very interesting and clinically relevant as *E. coli* is the most commonly encountered ESBL from community-acquired infections. More in-depth surveys with larger sampling and collaborations with other hospitals would generate more attractive ideas.

## Data Availability Statement

The raw data supporting the conclusions of this article will be made available by the authors, without undue reservation.

## Ethics Statement

Ethical review and approval was not required for the study on human participants in accordance with the local legislation and institutional requirements. Written informed consent to participate in this study was provided by the participants’ legal guardian/next of kin.

## Author Contributions

MK conceived and designed the study. RM, RB, and IS performed the experiments and analysis. FL, ID, and YA contributed with data and analysis. MK wrote the manuscript, with contributions and comments from all authors. All authors contributed to the article and approved the submitted version.

## Conflict of Interest

The authors declare that the research was conducted in the absence of any commercial or financial relationships that could be construed as a potential conflict of interest.

## Publisher’s Note

All claims expressed in this article are solely those of the authors and do not necessarily represent those of their affiliated organizations, or those of the publisher, the editors and the reviewers. Any product that may be evaluated in this article, or claim that may be made by its manufacturer, is not guaranteed or endorsed by the publisher.
